# New SI and precision measurements: an interview with Tianchu Li

**DOI:** 10.1093/nsr/nwz211

**Published:** 2020-02-25

**Authors:** Jin Wang

**Affiliations:** Professor of the Wuhan Institute of Physics and Mathematics, Chinese Academy of Sciences

## Abstract

On 13–16 November 2018, the 26th General Conference of Weights and Measures (CGPM) was held in Paris. The conference adopted Resolution A on ‘Revision of the International System of Units (SI).’ According to Resolution A: four of the SI basic units, namely kilograms, amps, kelvin and mole, are defined by the Planck constant h, the basic charge constant e, the Boltzmann constant k and the Avogadro constant N_A_, respectively. This establishes the basic quantities and units in SI on a series of constants. The new SI was officially launched on 20 May 2019. This is the most significant change and a milestone in the history of metrology since the Metre Convention was signed in 20 May 1875. Professor Tianchu Li, an academician of the Chinese Academy of Engineering, has been working on time and frequency standards for 37 years. In this interview, Prof. Li reviews the quantization and constant evolutions of the second and meter, and introduces the redefinitions of ampere, kelvin, kilogram and mole, and their significance for precision measurements.

## QUANTIZATION OF BASIC UNITS OF METROLOGY

**NSR:** What is metrology?

**Li:** I would like to give an example first. In 2012, *Nature News* reported the experimental results of a neutrino superluminal propagation. A bunch of neutrinos propagated from the European Nuclear Center in Switzerland to the Gran Sassoo National Laboratory in Italy 60 ns faster than the light in 730-km distance. But, a month later, it was found that the unusual experimental results were caused by a time measurement error. This shows that measurement plays a very important role in scientific research. The measurement must be ensured so that the measured values at different locations, different times, and measured by different methods are accurate and consistent. If not it will subvert the entire foundation of physics.

Metrology is originated from trade exchange, formed in the interchangeable demand in mass production in the Great Industrial Period, and developed in scientific research and defense industry. The modern metrology work began with the Metre Convention (MC). Based on the unified Metric System in French Revolution, as well as the British Industrial Revolution, 17 countries signed the MC on May 20, 1875 (World Metrology Day). China also joined the MC in 1976. The highest agency of the MC is the General Conference of Weights and Measures (CGPM), the second level is the International Committee of Weights and Measures (CIPM) and the third one is the International Bureau of Metrology (BIPM).The BIPM is a technical institute located in Paris and responsible for preserving the base standards, coordinating the comparisons and traceability of the base standards. Dedicated National Measurement Institutes (NMIs) are established in most countries around the world.

Metrology is the science of measurements, but these measurements are traced to the primary standards. If all measurements are traced back to a recognized primary standard, the measurement and metrology system is guaranteed to be uniform. Now, all countries in the world use the uniform metric standards. From these standards, through an uninterrupted traceability chain, end-user measurements can be traced back to the primary standards. In addition, the BIPM organizes a series of comparisons to ensure the normal operation of each traceability chain and each base standard. Most countries in the world have joined in the MC, and one of the largest contributions of the MC is the establishment of a unified International System of Units (SI).

**Figure:**
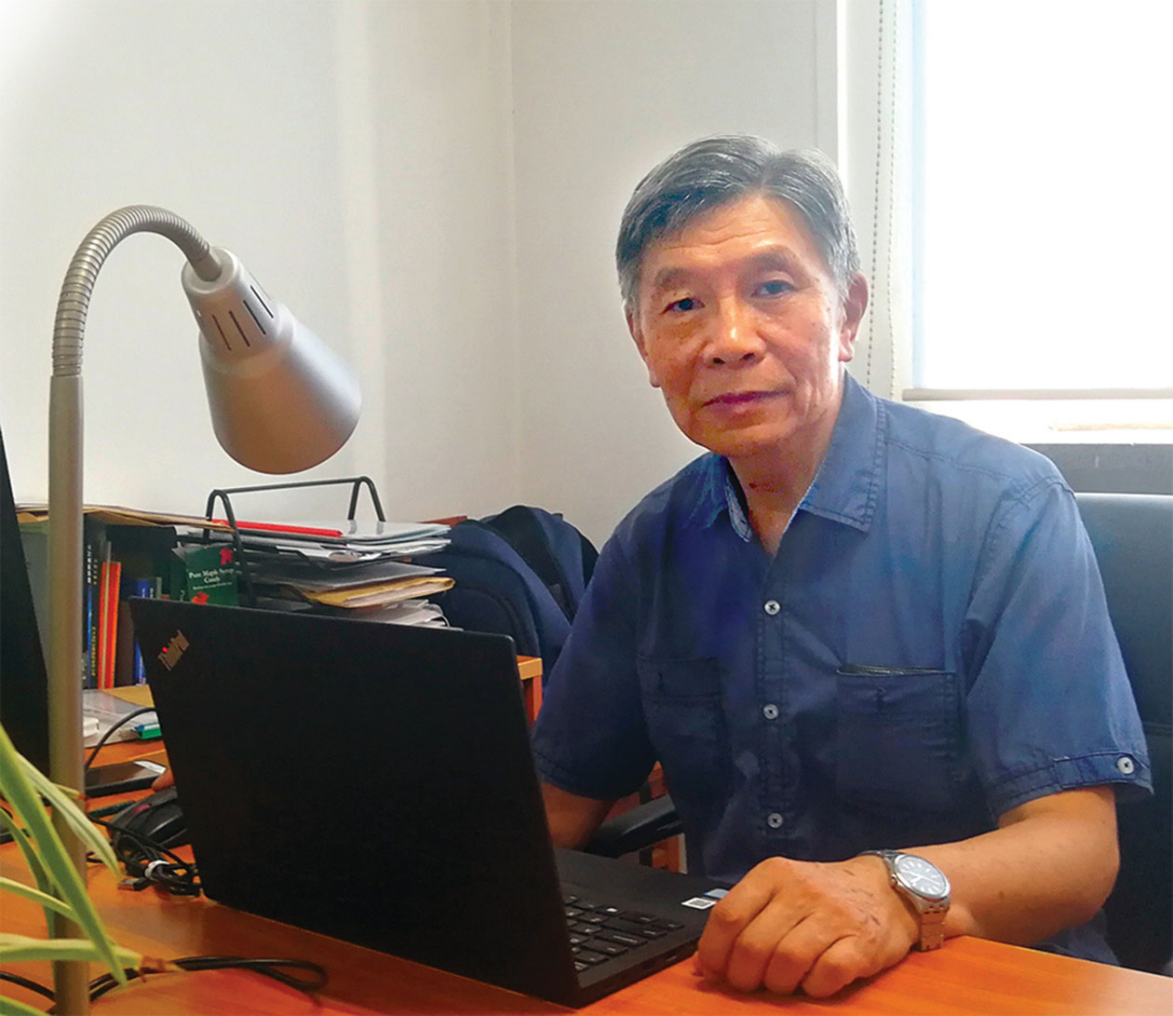
Tianchu Li, professor of NIM and well-known scientist in time and frequency standards *(Courtesy of Jin Wang)*.

**NSR:** What are the basic units in the International System of Units?

**Li:** The SI began in 1795. After continuous addition, improvement and molding, seven basic units were formed in 1971. All other physics quantities can be derived from these seven basic units. The seven basic units are unit of time—second (s), unit of length—meter (m), unit of mass—kilogram (kg), unit of electric current—ampere (A), unit of thermodynamic temperature—kelvin (K), unit of amount of substance—mole (mol) and unit of luminous intensity—candela (cd). Among them, the unit of amount of substance is a semi-physics and semi-chemistry unit, and the luminous intensity is somewhat special, depending on the human visual coefficient. I will mainly talk about the definitions of the first six units today.

**NSR:** When did the idea of the quantization definition of the basic unit originate?

**Li:** The earliest promoters of the quantization basic units were M. Planck and J. C. Maxwell. M. Planck once said ‘…with the help of fundamental constants, we have the possibility of establishing units of length, time, mass, and temperature, which necessarily retain all times and civilisations…throughout the universe.’

**NSR:** What happened to this SI revision?

**Li:** In the initial development stage of metrology, the definitions of the seven basic units depended on the prototype standards. For example, the length unit is defined as the distance between two engraving lines on the International Prototype Meter (IPM), and the mass unit is defined as the mass of the International Prototype Kilogram (IPK). After the middle of the twentieth century, with the development and maturity of quantum physics, it became a trend that the definitions of basic units changed from the prototypes to the quantum standards. Both time and length units have already been quantized, and their re-expressions based on constants were implemented in the SI revision in 2018. The main results of the 26th CGPM are the redefinitions of the four units, kilogram, ampere, kelvin and mole with constants.

## QUANTIZATION DEFINITION OF THE SECOND

**NSR:** When did the quantized definition of the second happen?

**Li:** Human understanding of time began with astronomy observations. The Earth's rotation-based Universal Time (UT) and the Earth's revolution-based Ephemeris Time are called Astronomical Time. The Greenwich Observatory officially began the release of AT on October 1, 1871. In 1955, P. Essen and J. Parry of the National Physical Laboratory (NPL) of the United Kingdom developed the first cesium atomic clock. In the same year, the US Naval Observatory and the NPL collaborated to measure the frequency of cesium referred to Astronomical Time as 9 192 631 770 Hz. Although the accuracy of that measurement was only 10^−9^, it was a very important milestone, and it marked a new definition that emerged from an old one. In 1967, the definition of an astronomical second was officially abolished in the 13rd CGPM, and 1 second was defined as the time of 9 192 631 770 cycles of radiation of the hyperfine level of Cs-133 atoms. Since then, the definition of the second has moved from astronomy to physics measurement.

**Figure:**
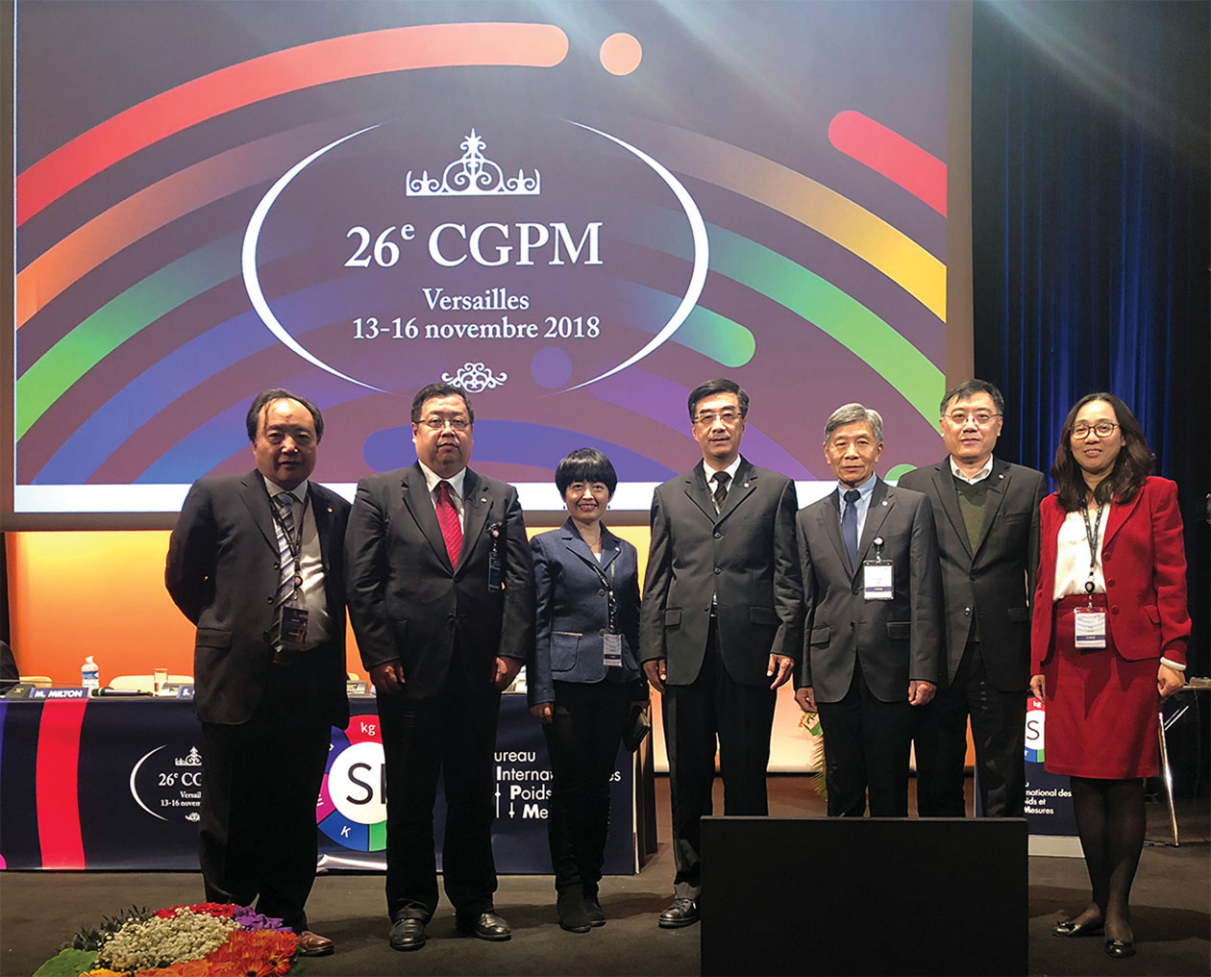
Tianchu Li (third from right) in the 26th CGPM with the delegation of China *(Courtesy of Tianchu Li)*.

The first cesium clock realized by the NPL is a primary standard clock. Since then, the primary standard clocks have evolved continually. In 1995, France realized the first laser-cooled cesium fountain clock. In 2010, China realized the cesium fountain clock NIM5, which passed the international review and participated to steer the International Atomic Time (TAI) from 2014. Now, China is developing the second standard fountain clock NIM6, and its initial uncertainty has reached 6 × 10^−16^.

**NSR:** How is international standard time generated and released?

**Li:** The primary standard clocks alone are not enough, and it is also necessary to carry out time-keeping. A weighted average of time-keeping clocks generates Atomic Time Scale, which is very reliable, because it is produced by a set of clocks instead of one clock.

At present, there are about 80 time-keeping laboratories and 500 clocks in the world participating in the TAI Cooperation led by the BIPM. The weighted average of the data of 500 clocks produces the Free Atomic Scale (EAL, Echelle Atomique Libre). EAL is both reliable and stable, but not accurate enough. Countries with primary standard clocks report their data to the BIPM from time to time to steer, or correct, the EAL, and the corrected EAL is the TAI.

After running for a period, the TAI can deviate from the astronomical UT. The International Earth Rotation Service in France is responsible for measuring the difference between the TAI and the astronomical UT1. When the time difference is approaching 0.9 seconds, a notice is issued, and a leap second is operated worldwide at the same time. The TAI corrected by a leap second is Coordinated Universal Time (UTC), which is now universal time worldwide.

The UTC is fed back to the time-keeping laboratories in the form of time bulletin, Circular T, every 30 days. Based on the difference between the feedback data from Circular T and the originally reported data, each laboratory corrects its own time and produces the more accurate local atomic time UTC(k), where k is the abbreviation of the laboratory. For instance, to date, there are three Chinese laboratories contributing to the EAL: UTC(NIM), UTC(NTSC) and UTC(BIRM) are the local atomic times realized by the National Institute of Metrology in China (NIM), the National Time Service Center of the Chinese Academy of Sciences (NTSC) and Beijing Institute of Radio Metrology(BIRM), respectively.

## THE FIRST UNIT DEFINED BY CONSTANT-METER

**NSR:** When did the constant definition of the meter begin?

**Li:** From 1792 to 1799, J.-B. Delambre and P. Mechain, commissioned by the French Academy of Science, measured the arc length of Earth meridian from Dunkirk through Paris to Barcelona using the geodetic triangle method, and defined 1 meter as forty-millionth of the length of the Earth's meridian. Based on that measurement, the IPM was then made.

In 1892–1893, A. A. Michelson measured the wavelength of the cadmium spectrum against the IPM. In 1960, the 11th CGPM decided to abolish the IPM and redefine the meter using the wavelength of the Kr-86 spectrum lamp. Using the spectrum lamp as the light source of an interferometer, the length would be measured by the interferometer according to the new definition of the meter. This is the first quantization definition in the true sense.

Shortly after the invention of the laser, the frequency stabilized laser completely replaced the Kr-86 lamp. In 1972, K. M. Evenson re-measured the speed of light using wavelength standard with better uncertainty. In 1975, the 15th CIPM set the speed of light as an error-free constant. In 1983, the length unit, meter, was defined by the physics formula, *l* = *ct*, where *l* is the length, *c* is the speed of light in vacuum and *t* is the time. Thus, the meter became the first unit defined by physics constant.

**NSR:** Is there any relationship between the meter and the second?

**Li:** There are several ways to realize the unit of length. The most important one is to trace the laser frequency through the optical comb to the microwave frequency, which is traced directly back to the definition of the second. In this way, the definition of the second is transitioned to the optical wavelength, and the second is closely related to the meter.

The process of unit redefinitions of second and meter can be summarized as follows: the old definition of the unit is used to measure a constant, the constant is defined, and the unit is redefined based on this constant. The experimental measurement of the constant becomes one of the methods of realizing the new definition.

—Tianchu Li

## FOUR OTHER UNITS DEFINED BY CONSTANTS

**NSR:** How were the four units of ampere, kelvin, kilogram and mole redefined in the 26th CGPM?

**Li:** The old definition of the ampere was very complicated and difficult to realize in practice. The new definition of the ampere followed the path of the meter and the second. First, the electronic charge is selected as the basic charge *e*, and its value is fixed as an error-free constant. The electron charges (basic charge) passed per second is called current, *I* = *ne/t*, where *I* is electric current, *n* is an integer and t is time. The physics concept is very clear, that is the *n* = 1/*e* charges passed per second is called 1 ampere. At present, the practical realization of the ampere is still using Ohm's law, by measuring the voltage and the resistance to calculating the current. The realization methods of voltage and resistance have been quantified already.

The old definition of the kelvin is the three-phase point (TPP) of water, which sets the temperature of the liquid, solid and gaseous three-phase states of water to 273.16 K. This seems very scientific and reasonable, but the TPP of water depends on its isotope composition; we can measure the composition of the isotopes of water, but cannot control them with state of art at present. The principle of the new definition of the kelvin is that the change in energy is equal to the Boltzmann constant multiplied by the change in temperature, Δ*E* = *k*Δ*T*, where *E* is energy, *k* is Boltzmann constant and *T* is temperature. The redefinition of the kelvin is based on the accurate measurement of Boltzmann constant.

The old definition of the kilogram was the IPK. The new definition is based on two formulas; the mass (*m*), Planck constant (*h*), the frequency of the matter wave (*ν*) and the speed of light in vacuum (*c*) can be related as: *E* = *hν* = *mc*^2^. The macroscopic mass, whose de Broglie-Compton frequency equals to *c*^2^/*h*, is called 1 kilogram. The realization method of the kilogram is achieved with a Kibble (Watt) balance, which translates the mechanical energy measurement into electrical energy measurement.

The mole is defined as the number of particles within a certain volume. Its redefinition depends on a certain number of particles, and no longer depends on the unit of mass—kilogram. The value of the Avogadro constant, *N*_A_ = 6.02214076 × 10^23^, is fixed as an error-free constant, and 1 mole accurately contains 6.02214076 × 10^23^ elementary particles, which is the fixed value of Avogadro constant expressed in unit of mol^−1^, 1 mol = 6.02214076 × 10^23^/*N*_A_.

**NSR:** What contribution did China make to the redefinition of SI?

**Li:** In the quantization definition of the second, there are nine countries involved in the steering of the TAI. However, from August 2016 to the present, only five countries including Germany, France, Russia, China and Italy joined the TAI correction. In the revolution of time and frequency, although China did not really participate at the beginning, the NIM5 fountain clock of NIM has contributed to the TAI since 2014.

In the redefinition of the kelvin, China has made a substantial contribution to the precise determination of the Boltzmann constant. The NIM accurately measured the Boltzmann constant using both the acoustic gas thermometer method and the noise-based thermometer method. These measurements are accepted by the Committee on Data for Science and Technology (CODATA).

NIM has developed a Joule balance like Watt balance, and carried out research works related to the realization of the kilogram.

The accurate determination of the Avogadro constant is critical in the constant definition of the mole. China participated in the international cooperation and comparison of Avogadro constant measurement. NIM measured the molar mass of concentrated silicon by two different methods, and the measurement results were formally adopted.

## THE INFLUENCE OF THE NEW SI ON THE WORLD

**NSR:** What is the overall influence of the new SI on metrology?

**Li:** The constant-based redefinition of the SI units is downward compatible, it has almost no effect on most end-users, and some adjustments may need to be done for the highest-end applications. However, the redefinition is divorced from the real prototype standards, the traceability chain is shorter, the accuracy is better, the full scale is covered, and the method of realization is open. The definition is unique, as technology advances it can be realized with new processes and new technologies.

**NSR:** From your professional perspective, what is the specific impact of the redefinition of seconds?

**Li:** The new definition of the second facilitates the flattening of time service. For example, the original time and frequency are delivered through transfer-clocks step by step from the

Among the four units of electric current, thermodynamic temperature, mass and amount of substance, the concepts of ampere, kelvin, and mole are clear. While the kilogram is defined based on de Broglie-Compton frequency, it could be a little difficult in scientific popularization even for the lower classman in the universities.

—Tianchu Li

base standard to the end-users. Now, the GPS, BEIDOU and GLONASS perform the time service function all over the world. The satellite time service is a flat, one-step transfer process which shortens the tracing link of time and frequency with better accuracies.

The atomic time is conducive to unify the interchangeability of mass industrial manufacturing. For example, China produces a shaft and wants to use a Swedish bearing. How do we ensure that the Swedish bearing can be mounted on the Chinese shaft without any modification? A micrometer could be used to measure the Chinese shaft, a Swedish micrometer was used to measure the Swedish bearing, a gauge block then could be used to calibrate the Chinese micrometer, and then the gauge was shipped to Sweden to calibrate the Swedish micrometer. This practice could certainly work, but it is not the best at all. The current practice is to use a laser, which traced to time, to measure micrometer in China. In Sweden the same is done. As the times of Sweden and China are unified to UTC, it is very reliable to ensure that the length measured in China is consistent with that measured in Sweden, and the shaft in China and the bearing in Sweden are completely interchangeable.

**NSR:** What is your outlook for the redefinition of seconds?

**Li:** The stability of a clock is inversely proportional to its nominal frequency. The frequency of light is 10^5^ higher than that of microwave, so the optical clock has better stability and uncertainty potentials. We noticed that after 10 years of development, the stabilities of optical clocks are much better than that of cesium fountain microwave clocks. At present, the uncertainties of the best fountain clocks in the world are 2 ∼ 4 × 10^−16^, while the uncertainty of the ytterbium optical clock was 1.4 × 10^−18^ in 2018, and the stability of the aluminum ion optical clock was 9.4 × 10^−19^ in 2019. The International Consultative Committee for Units (CCU) will officially summarize the last redefinitions of the four base units, and launch the study on redefinition of the second.

**NSR:** What is your overall view of redefinition of SI?

**Li:** The 26th CGPM redefined the base units based on seven constants. Some constants are true fundamental physics constants, and others are not. The time unit—second—is now based on the cesium frequency constant, which is not a strict constant. The CGPM named these seven constants as ‘defining constants’, and published their numerical values.

